# Changes in the optic nerve head induced by horizontal eye movements

**DOI:** 10.1371/journal.pone.0204069

**Published:** 2018-09-18

**Authors:** Won June Lee, Yu Jeong Kim, Ji Hong Kim, Sunjin Hwang, Seung Hak Shin, Han Woong Lim

**Affiliations:** 1 Department of Ophthalmology, Hanyang University Hospital, Seoul, Korea; 2 Department of Biomedical Engineering, Hanyang University College of Medicine, Seoul, Korea; University of Copenhagen, DENMARK

## Abstract

**Purpose:**

To investigate the effect of eye movement on the optic nerve head (ONH) using swept-source optical coherence tomography (SS-OCT), and to measure the degree of ONH changes.

**Methods:**

We enrolled 52 healthy subjects, 20 to 40 years of age, and performed a prospective observational study. Both ONH and macula were imaged simultaneously using wide volume scan of the SS-OCT in the primary and different gaze positions. Horizontal eye movements were used to obtain OCT images in abducted and adducted eyeball positions. Multilateral 3-dimensional registration was used to process and analyze the images to measure the degree of ONH changes.

**Results:**

The mean axial length (AXL) was 25.73 ± 1.42mm and the mean spherical equivalents was -4.49 ± 2.94 D (The proportion of high myopia was 39.4%). Significant morphologic changes were observed in the ONH during both abduction and adduction. In abduction, the overall ONH tissues were elevated, and the mean area of elevation was 115,134 ± 9,424 μm^2^ (p<0.001). In adduction, the mean areas from two perspectives, which were nasal or temporal, and peripapillary tissues or optic nerve cupping were 95,277 ± 73,846 μm^2^, 34,450 ± 44,948 μm^2^, -108,652 ± 91,246 μm^2^, and -30,581 ± 46,249 μm^2^, respectively. Elevation in abduction (overall, nasal cup segment, and temporal cup segment; R = 0.204, 0.195 and 0.225, p = 0.038, 0.047 and 0.021, respectively) and elevation of nasal peripapillary segments in adduction were positively correlated with AXL (R = 0.346, p<0.001).

**Conclusion:**

We found significant morphologic changes in the ONH in both abduction and adduction and these changes were associated with AXL. Considering these morphologic changes as physical properties, it allows a better understanding of the biomechanical characteristics of the ONH.

## Introduction

The optic nerve is a paired cranial nerve that transmits all visual information from the retina to the brain.[1, 2] The optic nerve is the main lesion of the optic neuropathies including glaucomatous optic neuropathy and the beginning of this, the optic nerve head (ONH), is a unique part of the central nervous system that can be examined directly with appropriate optical equipment and lenses. With the advancement of optical coherence tomography (OCT), it became possible to observe ONH non-invasively. [3–5] Many studies have evaluated the static structure of the ONH using OCT, especially in relation to glaucoma.[6–8]

The concept that eye movement can cause strain on the optic nerve was suggested in the early 19^th^ century[9], and several recent studies reported the ONH change according to the eye movement using OCT. One group recently has shown that peripapillary tissue deformation occurs during horizontal ductions using OCT in healthy participants, in those with anterior ischemic optic neuropathy, in those with papilledema,[10] and in those with optic ONH drusen.[11] Another study demonstrated that adduction but not abduction was associated with peripapillary tissue displacement.[12] We reported that the volcano-like morphological change occurred in the ONH during abduction in highly myopic patient with phosphenes.[13] Because observing structural changes of the ONH can help to infer the strain on the ONH following eye movement, evaluation of ONH changes is important; however, as mentioned above, the research remains controversial about the morphologic changes that are induced by the eye movement.

In this study, we investigated the effect of the horizontal eye movement on the ONH using swept-source OCT (SS-OCT) and measured the degree of ONH changes to evaluate correlations with the axial length (AXL).

## Material and methods

This study was approved by the Institutional Review Board of Hanyang University Hospital with informed consent obtained. The study design followed the tenets of the Declaration of Helsinki for biomedical research.

### Participants

This study included a total of 52 healthy subjects that ranged between 20–40 years (33 males, 19 females). We recruited subjects who voluntarily consented after fully explaining the study. All participants underwent a comprehensive ophthalmologic examination, including best-corrected visual acuity (BCVA), refraction, slit-lamp examination, dilated fundoscopy, AXL measurement, and stereoscopic optic disc photograph. Axial length measurement was obtained using the IOLMaster (Zeiss Humphrey System, Dublin, CA) following the procedures recommended by the manufacturer in the IOLMaster user’s manual.

Exclusion criteria were as follows: (1) any retinal disorder or optic nerve disorders including glaucoma at the comprehensive ophthalmologic examination including macular and ONH scan in primary position, (2) any abnormal ocular motility dysfunction finding with physical examination (duction and version, cover uncover test) by strabismologists, (3) neurologic diseases, (4) diabetes, and (5) previous ocular or periocular surgery.

### Image acquisition procedures

Participant images were acquired using deep range imaging swept source OCT (DRI OCT-1 Atlantis; Topcon, Tokyo, Japan), with 12-mm horizontal and 9-mm vertical wide-field scans. The DRI OCT-1, which is available for a 1,050 nm wavelength light source, was used to obtain volume scans and the technique included simultaneous ONH and macula scans with an internal-fixation target (which was displayed in OCT machine screen during the image acquiring) between the macula and ONH. This approach provided us with advanced 3-dimensional (3D) volumetric layer detection algorithms, which were effective for 3D analysis of the ONH morphology.

The macula and retinal nerve fiber scans were performed in each eye, respectively, to demonstrate that there were no ONH and macula disorders. We excluded the subjects who showing any retinal disorder or optic nerve disorders including glaucoma at the comprehensive ophthalmologic examination including macular and ONH scan in primary position. Wide-volume scans of the ONH and macula in the primary position were performed in both eyes with an internal-fixation target which was placed between the macula and ONH. Using this internal-fixation target, we were able to avoid unexpected prominent adduction in the volume scans whose fixation target was the ONH. We could also use the ONH and the macula as our major scanning targets, which was important for analyzing changes in the ONH.

After scanning images of the primary position, subjects were instructed to rotate their head to the right-side by approximately 30° which was confirmed by an examiner with a goniometer and the eyes keep looking straight ahead (into the OCT machine). We obtained the wide volume scan of the right eye in the adducted position and also the scan of the left eye in the abducted position (Pannel A in S1 Fig). Then, we instructed the subjects to rotate their head to the left-side by approximately 30°, and performed the same scan of the right eye in the abducted position and the left eye in the adducted position (Pannel B in S1 Fig).

### Image-processing procedures (3D registration)

Each volume scan was composed of 256-packaged gray-scale B-scans images, and the resolution of each B-scan image was 1024*512 pixels. Analysis of biomechanical changes in the ONH was implemented by self-produced visual C++, Visual Studio Community software (Version 2015; Microsoft, Redmond, WA, USA). Multilateral viewpoints were composed of axial, sagittal, coronal views with 3D-registration. This was processed with the software for accurate 3D analysis (S2 Fig). Before quantitative measurement, we used the 3D-registered images to verify the comprehensive tendency of ONH biomechanical changes.

**Fig 1:**
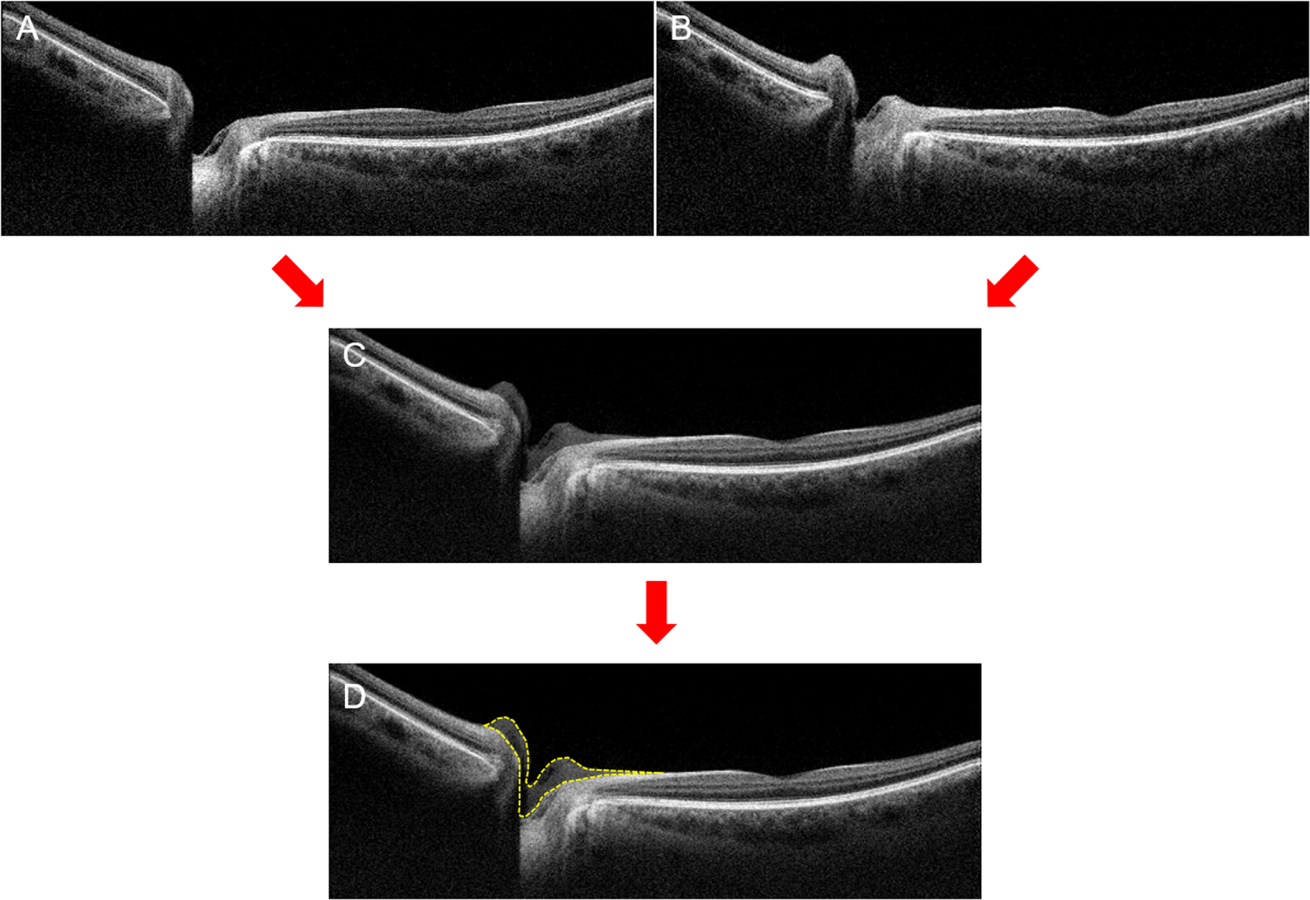
Image-processing flow from the optical coherence tomography (OCT) B-scan images to calculating the area of optic nerve head morphological changes. The OCT B-scan images in central gaze (A) and horizontal gaze (B). (C) Superimposed OCT images with the image-movement functions from every direction and using image-tilting. (D) Quantitative measurement performed with manually-set pointers on optic nerve head tissues to form each measurement area. (For well-visualizing, original B-scan images were presented).

The overall processing flow from loading the packages of the original B-scan images to calculating the area of ONH morphological changes is presented in 3 stages. First, dedicated image-processing software loaded the bundled gray-scale-based ONH images obtained from participants. After the original bundled images were loaded, they were 3D processed using a reconstructing function to minimize unexpected errors during superimposition and area-based ONH measurement (which can show the axial, sagittal, coronal views simultaneously; S2 Fig). Then, from the filtered and reconstructed images, a green cross hair was placed at the ONH and the foveal dimpling, which were the focus point for superimposition. With regard to ONH, we set the center of it as Bruch’s membrane opening (BMO)’s center based on an approximation. Second, image transformation by superimposition before the measurements was performed with the image-movement functions from every direction and using image-tilting (including rotation). We referred to the 3D-registered images during superimposition and confirmed the static tissues, without noticeable movement (ex. peripheral retina, retinal pigment epithelium layer), which were then considered the reference plane. Images were compared between the primary position and the horizontal gaze position, and relatively more precise image-matching based on the reference plane was possible when using this approach (3D registration; S1 Video). Third, quantitative measurement was performed with manually-set pointers on ONH tissues to form each measurement area (2D measurement). The boundary of differences was outlined with pointers, and the areas of differences were automatically calculated (Fig 1D).

### ONH changes analysis (2D measurement)

**Fig 2:**
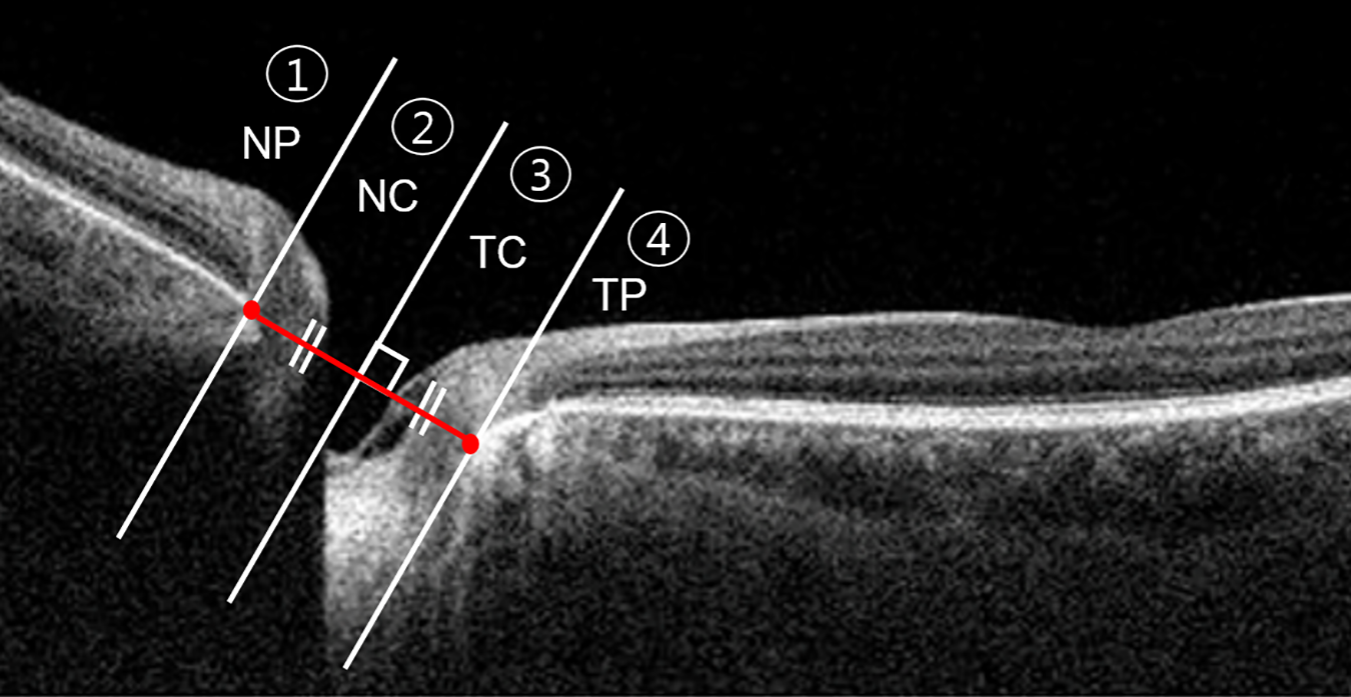
Schema of optic nerve head (ONH) segmentation method. A line connecting the two Bruch’s membrane openings (BMOs) was drawn at the primary position. Then vertical line dividing this line equally was drawn and the nasal and temporal sides were distinguished based on this vertical line. Each nasal or temporal-sided segment was classified into 2 groups based on BMO of ONH. (NP = nasal peripapillary segment, NC = nasal cup segment, TC = temporal cup segment, TP = temporal peripapillary segment).

**Fig 3:**
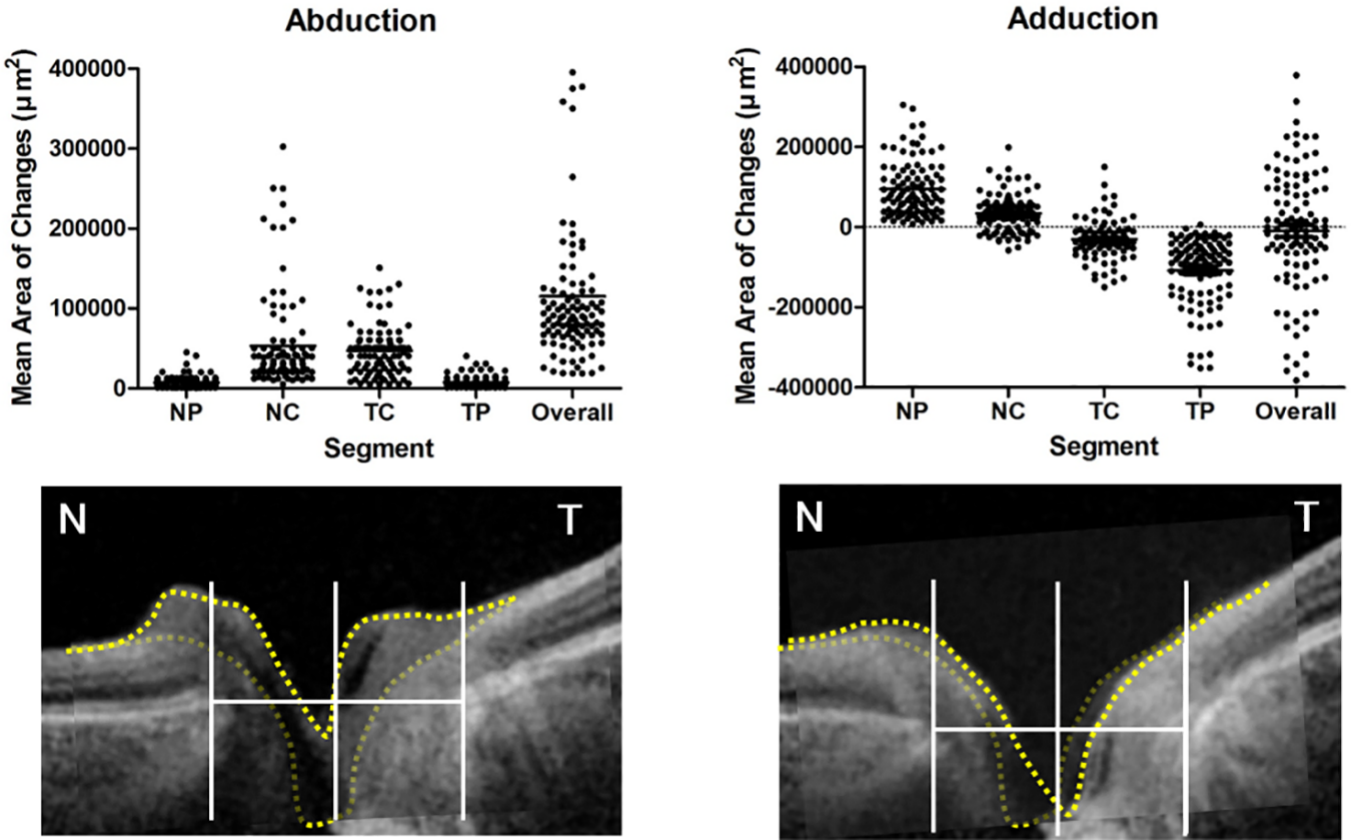
Overall and segmental optic nerve head (ONH) changes in abduction and adduction. The distribution of ONH changes were provided. Bright yellow dotted line is configuration in horizontal eye movements, and faded yellow dotted line is configuration in primary position. (NP = nasal peripapillary segment, NC = nasal cup segment, TC = temporal cup segment, TP = temporal peripapillary segment, N = nasal, T = temporal).

The superimposed continuous packages of B-scan images were imported into the software. We selected the scan, which represented the center of the ONH. After interactively matching the primary and horizontal duction position images, boundary differences were clarified by lines that were composed of pointers (Fig 1D). Four segments of the ONH structure were newly defined. A line connecting the two BMOs was drawn at the primary position. Then vertical line dividing this line equally was drawn and the nasal and temporal sides were distinguished based on this vertical line. Each nasal or temporal-sided segment was classified into 2 groups based on BMO of ONH (Fig 2). Each independent area from the difference of the boundary lines was calculated with this method (Yellow dotted lines in Fig 3). The area was extracted and calculated using self-produced software (2D measurement). In this analysis, static ONH tissues and macular areas were found to be an important reference plane. To determine interobserver reliability, 2 independent observers (H.W.L. and J.H.K.) measured the “area of difference” using the method described.

Optic disc size was evaluated using the ‘disc area’ parameter in the wide volume SS-OCT map which was calculated with embedded software. Optic disc tilt was identified by the ‘tilt ratio’, defined as the ratio between the longest and shortest diameters of the optic disc.

### Statistical analysis

Statistical analyses were performed using SPSS for Windows version 20.0 (SPSS, Inc., Chicago, IL, USA). Because our study was designed for normal physiology, each independent eye was considered to be a standardized unit of analysis.[14, 15] We repeated statistical analyses using a generalized estimating equation (GEE) model to account for interocular correlations within participants. Based on the hypothesis that the primary position had zero change, the areas of ONH changes were compared to the primary position using 1-sample t-tests. Linear regression analysis was performed to assess the relationship between ONH change mean areas and other parameters including AXL, disc area, tilt ratio, and intraocular pressure. A value of P<0.05 was considered statistically significant.

## Results

One hundred four eyes from fifty-two subjects were included in this study: 33 males and 19 females (age range, 20–40 years; mean, 25.4 years). The AXL ranged from 22.56mm to 29.13mm with an average of 25.73 ± 1.42mm. The refractive errors ranged from -9.5 diopters (D) to +2.0 D with an average of -4.49 ± 2.94 D in spherical equivalent (SE). The proportion of high myopia was 39.4%, which was defined as SE of -6.0 D or less or an AXL of 26.5 mm or more in this study. Clinical demographics of enrolled subjects were shown in Table 1. Our image scanning procedure was applied in all horizontal gazing positions with no experimental exceptions because the image acquisition procedure was well-tolerated by all subjects.

**Table 1:** Clinical demographics of enrolled subjects.

	Mean values ± Standard deviations
Age (years)	25.38 ± 2.89
Gender (Male/Female)	33/19
IOP (mmHg)	15.17 ± 2.64
Axial length (mm)	25.73 ± 1.42
Spherical equivalent (D)	-4.49 ± 2.94
High myopia^†^ (%)	41 eyes (39.4%)
Disc Area (SS-OCT)	1.96 ± 0.40
Optic disc tilt ratio*	0.79 ± 0.11

^†^High myopia refers to a spherical equivalent of -6.0 D or less or an axial length of 26.5 mm or more.

*Optic disc tilt was identified by the tilt ratio, defined as the ratio between the longest and shortest diameters of the optic disc.

SS-OCT; swept source optical coherence tomography.

**Fig 4:**
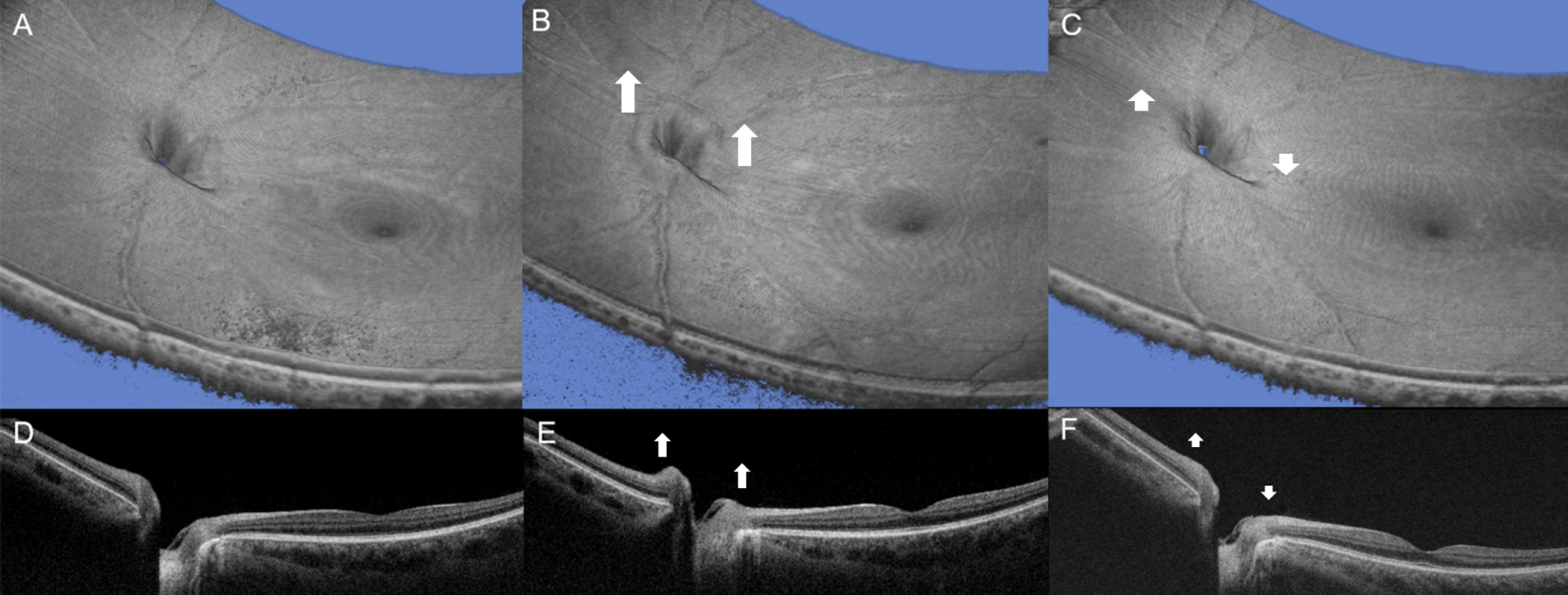
Optical coherence tomography (OCT) images showing optic nerve head changes. 3-dimensional OCT images and B-scan OCT images in the primary position (A & D), abducted position (B & E), and adducted position (C & F). (For well-visualizing, original B-scan images were presented). White arrows indicated the morphological changes of optic nerve head according to the horizontal eye movements.

Most of the eyes showed morphological changes to the ONH during horizontal eye movements. Compared to the ONH in the primary position (Fig 4A and 4D), overall ONH tissue in all four segments was elevated in abduction (Fig 4B and 4E). Different changes to ONH were observed during adduction; Nasal-sided peripapillary tissues were elevated, and the temporal-sided were depressed (Fig 4C and 4F). The change in the optic nerve cupping was affected by peripapillary tissue adduction.

The difference in the linear parameters of the primary position and abduction or adduction was extracted as area in square micrometer units (μm^2^) and was determined by self-produced software (Table 2 and Fig 4). The mean ONH change values, according to horizontal gazing, are given in the Table. The mean area of overall elevation in abduction was 115,134 ± 9,424 μm^2^ (p<0.001). These significant changes were observed in all four segments (nasal peripapillary, nasal cup, temporal peripapillary, and temporal cup segment; 7,267 ± 7,611 μm^2^, 53,259 ± 59,293 μm^2^, 7,505 ± 7,253 μm^2^, and 47,103 ± 30,358 μm^2^, respectively). In adduction, the mean areas from two perspectives, which were nasal or temporal, and peripapillary tissues or optic nerve cupping were 95,277 ± 73,846 μm^2^, 34,450 ± 44,948 μm^2^, -108,652 ± 91,246 μm^2^, and—30,581 ± 46,249 μm^2^, respectively.

**Table 2:** Mean areas of optic nerve changes from primary position.

Horizontal Gazing	Area	95% CI	P value*
Abduction			
Overall	115,134 ± 96,108	96,443 ~ 133,825	<0.001^†^
Nasal Peripapillary Segment	7,267 ± 7,611	5,786 ~ 8,747	<0.001^†^
Nasal Cup Segment	53,259 ± 59,293	41,728 ~ 64,790	<0.001^†^
Temporal Peripapillary Segment	7,505 ± 7,253	6,094 ~ 8,916	<0.001^†^
Temporal Cup Segment	47,103 ± 30,358	41,199 ~ 53,007	<0.001^†^
Adduction			
Overall	-9,506 ± 151,392	-38,948 ~ 19,936	0.523
Nasal Peripapillary Segment	95,277 ± 73,846	80,916 ~ 109,638	<0.001^†^
Nasal Cup Segment	34,450 ± 44,948	25,709 ~ 43,192	<0.001^†^
Temporal Peripapillary Segment	-108,652 ± 91,246	-126,397 ~ -90,907	<0.001^†^
Temporal Cup Segment	-30,581 ± 46,249	-39,576 ~ -21,587	<0.001^†^

Positive and negative values are elevation and depression respectively.

*One sample t test.

^†^Indicates a significant difference from the reference plane.

Data are presented as mean values ± standard deviations.

The relationship between ONH change areas and AXL is shown in Table 3. Elevation in abduction (overall, nasal cup segment, and temporal cup segment; R = 0.204, 0.195 and 0.225, p = 0.038, 0.047 and 0.021, respectively, GEE analysis p<0.001) and elevation of nasal peripapillary segments in adduction were positively correlated with AXL (R = 0.346, p<0.001, GEE analysis p<0.001). However, there were no significant correlations between AXL and temporal depression of peripapillary tissues, changes of optic nerve cupping in adduction. Also, there was no significant effect of optic disc size, disc tilt or intraocular pressure on the ONH changes according to the horizontal duction.

**Table 3:** Correlation coefficients of optic nerve changes on other parameters.

	Axial length	SE	Disc area	Tilt ratio	IOP
Abduction					
Overall	0.204*	-0.153	0.076	-0.003	-0.097
Nasal Peripapillary Segment	0.072	-0.036	0.052	0.024	-0.114
Nasal Cup Segment	0.195*	-0.158	0.039	-0.030	-0.075
Temporal Peripapillary Segment	0.080	-0.080	0.062	-0.043	-0.161
Temporal Cup Segment	0.225*	-0.147	0.137	0.054	-0.093
Adduction					
Overall	0.130	-0.086	0.003	-0.023	0.149
Nasal Peripapillary Segment	0.346**	-0.326**	-0.182	-0.148	-0.032
Nasal Cup Segment	0.113	-0.102	0.043	0.033	0.097
Temporal Peripapillary Segment	-0.081	0.102	0.058	-0.019	0.214
Temporal Cup Segment	-0.079	0.138	0.144	0.166	0.025

*,** Significant at 5% and 1% levels of significant respectively

SE; spherical equivalent, IOP; intraocular pressure.

## Discussion

Through 3D registration, we determined that horizontal eye movements, both adduction and abduction, structurally transformed the ONH. These changes included the overall elevation of the ONH on abduction in addition to nasal elevation and temporal depression of the ONH during adduction. We also found that elevation of the ONH on abduction and elevation of nasal ONH on adduction was significantly correlated with AXL.

Some studies have assessed peripapillary tissue deformations based on ductions. Using spectral domain OCT, Sibony reported that horizontal eye movements induce a relative “seesaw-like” shape deformation of the peripapillary basement membrane in normal subjects.[10] Whereas, Chang et al. reported that adduction but not abduction was associated with significant relative posterior displacement of the temporal peripapillary retinal pigment epithelium.[12] These effects have been determined, based on MRI and biomechanical evidence.[16] Similarly, Wang et al. reported temporal pulling and nasal compression in adduction and insignificant deformations of the ONH in abduction using OCT and in vivo strain mapping.[17]

Despite the results of these studies, the effects of horizontal eye movements on the ONH have not been clearly elucidated. The contradictory results from these studies might be due to the small number of participants. Furthermore, it is challenging to understand the overall morphological change tendencies that result from eye movements, track a reference plane and qualitatively measure peripapillary tissue deformation using 2D methods. Our previous case report based on disc photographs and 3D OCT images noted that remarkable changes to the ONH during abduction could be observed in high myope.[13] Based on these results, we were able to apply 3D OCT images to better understand changes in the ONH, which impacted our research approach.

Our method can evaluate morphological changes to the ONH far more accurately by comparing 3D planes (3D registration). This approach allowed us to use quantitative measurement, to recognize meaningful morphologic changes in the ONH by abduction and adduction. Moreover, precise superimposition of images was performed by comparing horizontal, axial, sagittal and 3D registered images (S1 Video).[18] Previous OCT studies primarily superimposed two images in 2D methods, and then defined reference plane and performed comparisons based on them. Also in our study, no clear reference plane was set up with a fixed anatomical landmark. Inaccuracy in the reference plane cannot be completely avoided in our study, but our approach is more objective and accurate because we evaluated the change in the ONH with a 3D-based reference plane.

The concept that eye movement can cause strain on the optic nerve and surrounding eye wall was suggested by Purkinje and Helmholtz in the early 19^th^ century.[9] Recently, Demer discussed in his MRI study paper that the optic nerve sheath which envelops the optic nerve is 9-fold more rigid than peripapillary sclera.[16] The material properties of optic nerve sheath itself and hydraulic stiffening [10, 19] or the contribution of dura[20] could be associated with the biomechanics of the ONH and these may have affected the results of this study.

**Fig 5:**
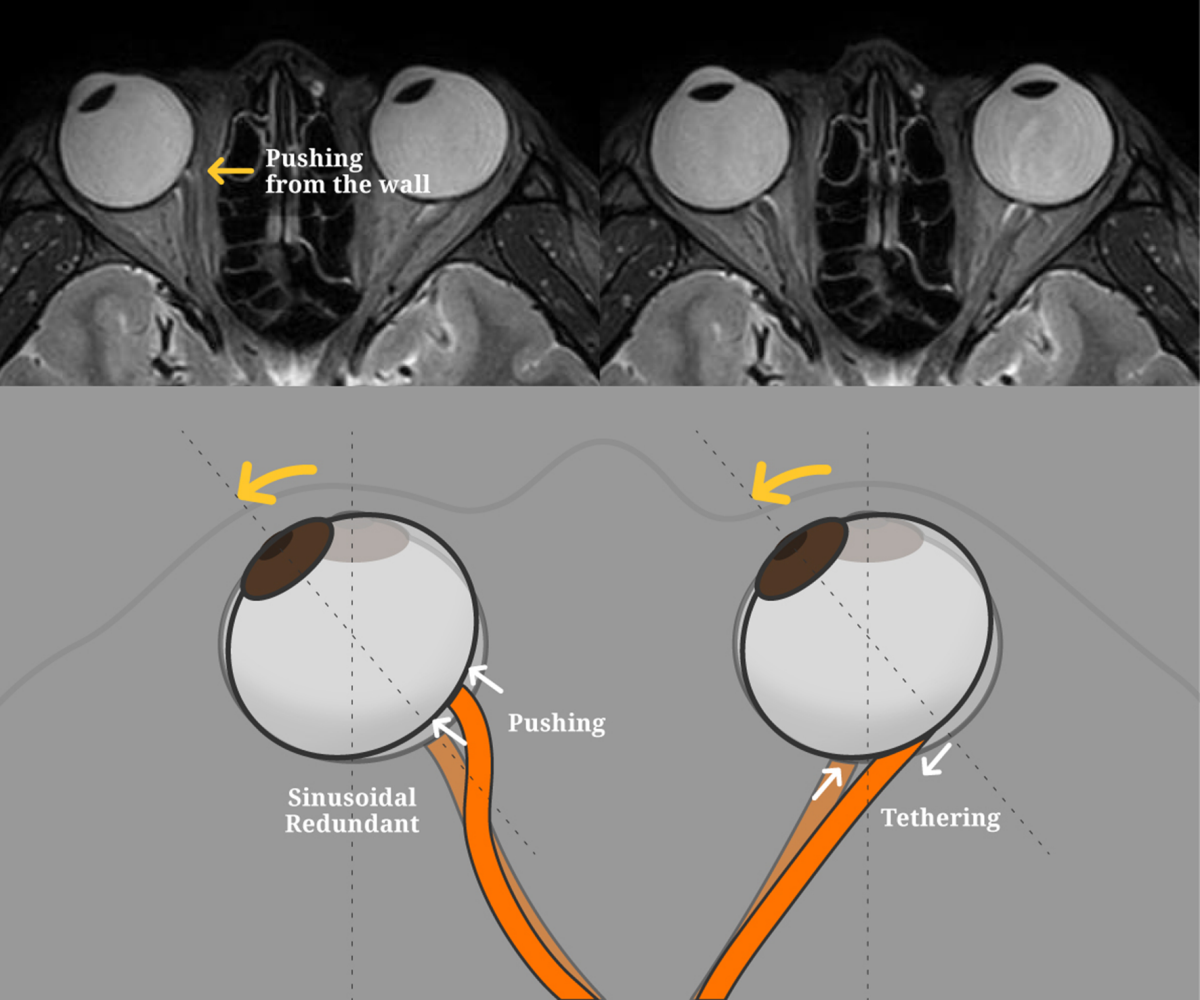
Schematic drawing with MRI images to illustrate our hypothesis. (Abduction) The ON becomes more redundant and sinusoidal during abduction. We hypothesized that the redundant ON in the abducted position would push the eyeball inward. In addition, it is presumed that the ON is moved to the medial side by abduction, and pressed by the narrow medial orbital space. As a result, the overall elevation of the ONH could occur in the abducted position. (Adduction) There is a stronger mechanical strain in adduction, especially for temporal peripapillary tissues. As a result, there could be elevation of the nasal side of the ONH and depression of the temporal side during adduction.

We found in this study that the overall elevation of the ONH occurred in the abducted position. Previous MRI studies have reported that the ON becomes more redundant and sinusoidal during abduction.[16, 21] We hypothesized that the redundant ON in the abducted position would push the eyeball inward. In addition, it is presumed that the ON is moved to the medial side by abduction, and pressed by the narrow medial orbital space (Fig 5). If the AXL is long due to myopia, these two possible effects would be greater.

Other OCT studies that assessed peripapillary tissue deformation based on horizontal duction reported that abduction was not associated with significant peripapillary displacement.[10, 12, 17] These abduction results are inconsistent with our study. A possible reason for these discrepancies is because the other studies performed OCT scans of the ONH where the internal-fixation target was placed approximately 15 degrees nasally to the centered ONH, which could decrease the effect of abduction. To overcome bias due to internal target position in the OCT scanner, Suh et al. offset the internal-fixation target to the point determined in preliminary scans in which subjects fixated 3 m distant target with the nonimaged fellow eye.[22] As a result, the seesaw-like shape deformation as that described by Sibony[10] was observed, which was different from our results. On the other hand, they also observed an anterior displacement of the optic cup during abduction, which was similar to our results. The main reason for the difference in the results seems to be the difference in the enrolled subjects. Subjects participating in this study had a high percentage of myopia, and many of them had high myopia (39.4%). Myopic eyes may be biomechanically and morphologically different from emmetropic eyes, which may show differences from the previous studies.

In this study, we found that there was elevation of the nasal side of the ONH and depression of the temporal side during adduction. These adduction results are similar to previous reports that used 2D analysis.[10, 12] The position, where the optic nerve joins the eyeball, is placed 3 to 4 mm to the nasal side of the posterior center of the eyeball.[23] The moving distance of the ONH in both directions’ horizontal eye movements is different even though the amount of angle is the same, thus the degree of the distance moved during adduction becomes longer compared to that of abduction. Therefore, there is a stronger mechanical strain in adduction, especially for temporal peripapillary tissues (Fig 5).

We found that both the overall elevation of the ONH on abduction and depression of temporal ONH on adduction were positively correlated with AXL. These morphological changes were correlated more with AXL than SE, suggesting that anatomical changes due to axial elongation might have affected the results. Anatomically, myopic eyes have thinner and less rigid sclera than nonmyopic eyes, and the ONH of highly myopic eyes (elliptical shape) travels more than that of emmetropic eyes when the eyes turn outward at the same angle.[14] Thus, in myopia, where the eyeball is relatively longer, the moving distance of the ONH becomes longer and the space between the optic nerve and EOM becomes narrower. Therefore, these physical properties explain the biomechanical relationship between prominent changes to the ONH and myopia.

This study has some limitations. First, we could not confirm the change of ONH according to the graded duction by only analyzing the abduction and adduction at the fixed angle of about 30 degrees. To reinforce the accuracy, the measurement center of the angle from the goniometer was determined individually from the perspective of each participant’s vertex (S1 Fig). Second, the potential for measurement error and large standard deviation due to variability of the reference plane existed because standardization and quantitative tracking of an absolute reference plane was difficult. Thus, we established reference planes in a more consistent way using 3D registration. Third, although we used 3D registration to minimize the errors in superimposition, mild torsions occur inevitably during the horizontal duction. We tried to control the torsional effect during the horizontal duction by rotating the OCT images during the 3D registration. However we could not control the stretching/compressing transformation of posterior pole which could be induced by the eye movement. Curvature of the posterior pole or distance between ONH and fovea could be changed during the horizontal duction and this can act as a bias when we superimposed the exactly same location in series of OCT scans. Fourth, the 3D registration was done by hand rather than a computer algorithm. This manual registration can cause errors. Future more advanced engineering support would make this study more sophisticated. Fifth, there was no control group that can determine statistically significant structural changes by calculating intersession deviation between 2 repeated measurements. Sixth, we only enrolled the young participants ranged between 20–40 years. Elasticity of ONH might be different depending on age. Further study comparing the ONH structural changes according to the age would be interesting. Last, we only evaluated the horizontal duction, not vertical duction. Further study evaluating more delicate ONH changes according to all directional eye movements would be needed.

In conclusion, our study demonstrated that there were morphologic changes to the ONH during both abduction and adduction and these changes were associated with the AXL. Considering these morphologic changes as physical properties, it allows a better understanding of the biomechanical characteristics of the ONH.

## Supporting information

S1 Fig The method of optic nerve head image acquisition during horizontal eye movement using optical coherence tomography (OCT). A. Clockwise head rotation of 30° from the baseline head position for imaging a right eye in adduction and left in abduction. B. Counterclockwise head rotation of 30° from the baseline head position for imaging a right eye in abduction and left eye in adduction. Subjects were instructed to rotate their head about 30° which was confirmed by an examiner with a goniometer. To reinforce the accuracy, the measurement center of the angle from the goniometer was determined individually from the perspective of each participant’s vertex.
(TIF)

S2 Fig Overview of self-produced visual C++, Visual Studio Community software. Multilateral viewpoints were composed of axial (A), sagittal (B), coronal (C) views with 3D-registration (D).
(TIF)

S1 Video Image-processing procedure with self-produced visual C++, Visual Studio Community software. The real image-processing procedure using in this study (3D registration process) was presented.
(AVI)

S1 Dataset Clinical and optical coherence tomography measurement data in enrolled healthy subjects. (XLSX)
