# Correction: Changes in the optic nerve head induced by horizontal eye movements

**DOI:** 10.1371/journal.pone.0216861

**Published:** 2019-05-09

**Authors:** Won June Lee, Yu Jeong Kim, Ji Hong Kim, Sunjin Hwang, Seung Hak Shin, Sukwoo Hong, Han Woong Lim

Dr. Sukwoo Hong should be included in the author byline. Dr. Hong should be listed as the sixth author, and Dr. Hong’s affiliation is 3: Department of Electrical and Biomedical Engineering, College of Engineering, Hanyang University, Seoul, Korea. The contributions of this author are as follows: Data curation, Formal analysis, Investigation, Methodology, Writing–original draft

The correct citation is: Lee WJ, Kim YJ, Kim JH, Hwang S, Shin SH, Hong S, et al. (2018) Changes in the optic nerve head induced by horizontal eye movements. PLoS ONE 13(9): e0204069. https://doi.org/10.1371/journal.pone.0204069

.
