# Supplementary figures and images for: Changes in the optic nerve head induced by horizontal eye movements
Source: PLoS One. 2018 Sep 18;13(9):e0204069. doi: 10.1371/journal.pone.0204069 (PMC6143247; doi:10.1371/journal.pone.0204069)

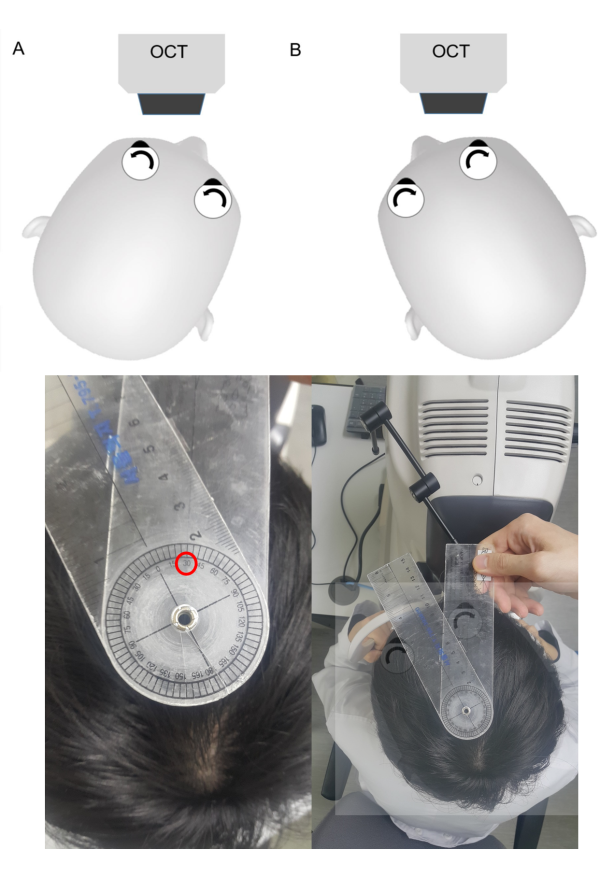

Supplement: S1 Fig — A. Clockwise head rotation of 30° from the baseline head position for imaging a right eye in adduction and left in abduction. B. Counterclockwise head rotation of 30° from the baseline head position for imaging a right eye in abduction and left eye in adduction. Subjects were instructed to rotate their head about 30° which was confirmed by an examiner with a goniometer. To reinforce the accuracy, the measurement center of the angle from the goniometer was determined individually from the perspective of each participant’s vertex. (TIF) [file pone.0204069.s001.tif]

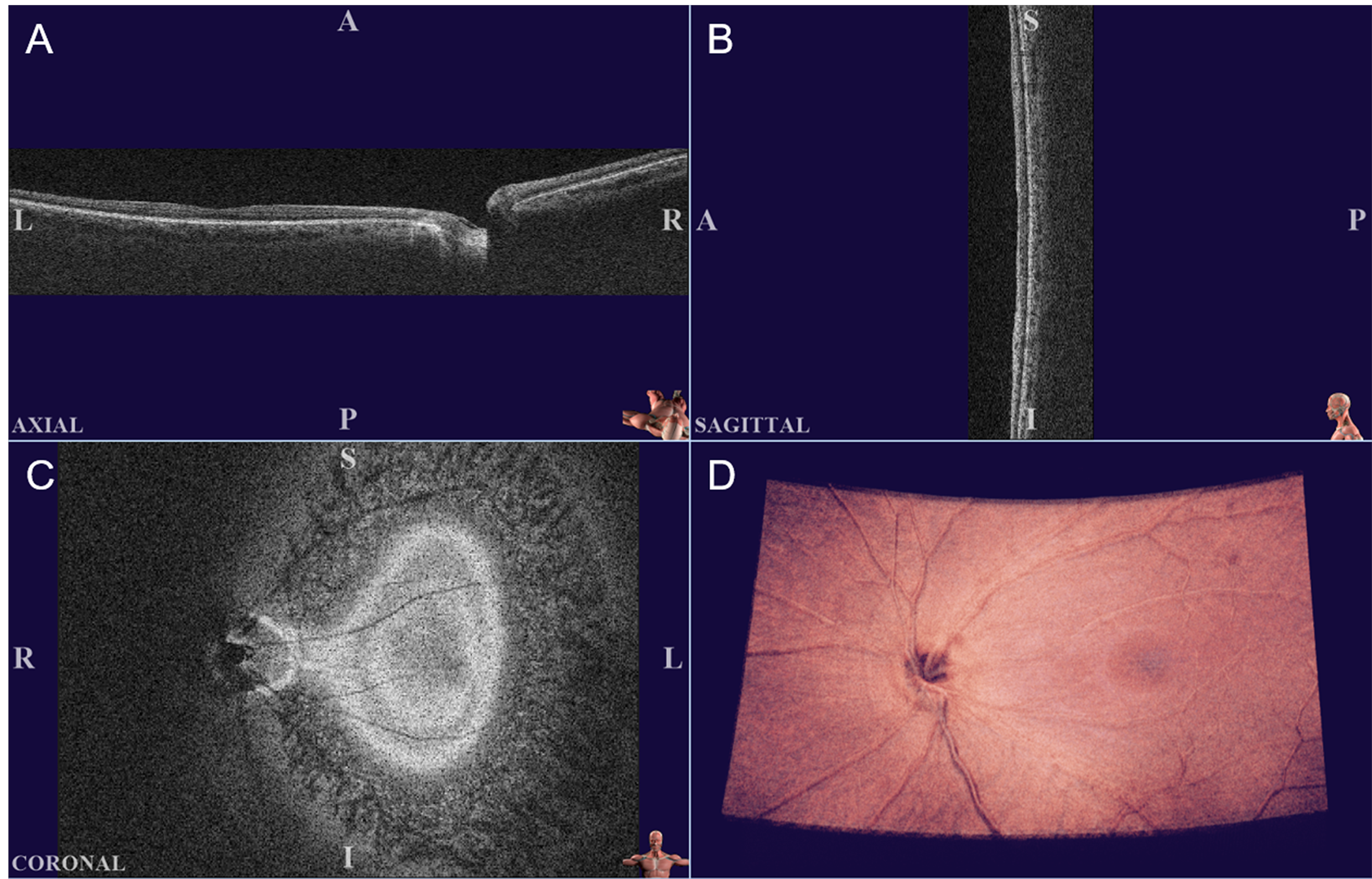

Supplement: S2 Fig — Multilateral viewpoints were composed of axial (A), sagittal (B), coronal (C) views with 3D-registration (D). (TIF) [file pone.0204069.s002.tif]
